# The complete chloroplast genome sequence of medicinal plant: *Dianthus chinensis* (Caryophyllaceae)

**DOI:** 10.1080/23802359.2020.1866453

**Published:** 2021-02-08

**Authors:** Zhen Yang, Xuhang Wang, Dong Wang, Panpan Xue, Ning Miao

**Affiliations:** a Key Laboratory for Bio-resource and Eco-environment of Ministry of Education, College of Life Sciences, Sichuan University, Chengdu, Sichuan, P. R. China; b PowerChina Huadong Engineering Corporation Limited, Hangzhou, Zhejiang, P. R. China

**Keywords:** Chloroplast genome, phylogenetic analysis, Caryophyllaceae, *Dianthus chinensis*

## Abstract

*Dianthus chinensis* is a medicinal plant. Its complete chloroplast genome sequence is 149,570 bp in length, containing 126 complete genes, including 84 protein-coding genes (84 PCGs), 8 ribosomal RNA genes (8 rRNAs), and 34 tRNA genes (34 tRNAs). The overall GC content of cp DNA is 34.1%, the corresponding values of the LSC, SSC, and IR regions are 34.0%, 29.8%, and 42.5%, respectively. Phylogenetic tree shows that *D. chinensis* is a sister to *D. longicalyx*.

The *Dianthus* genus consists of nearly 300 species native to Europe and Asia (Czerepanov 1973; Tang and Lu 1996). The blooms of *Dianthus chinensis* Linnaeus are five petaled. The petals are deeply notched, giving them a feathery or fringed appearance. *D. chinensis* is used as a popular garden plant over 2000 years. It is taken internally to treat acute urinary tract infections, urinary stones, constipation and failure to menstruate (Wang et al. 1998). It is applied externally to treat skin inflammation and swelling. The leaves are used in the treatment of hemorrhoids, lumbricoid worms, and venereal sores, while the flowers are used as an astringent, diuretic, hemostatic, resolvent, and vulnerary (Shin et al. 2012). However, the chloroplast genome of *D. chinensis* has not been reported. In this study, we assembled the complete chloroplast genome of *D. chinensis*, hoping to lay a foundation for further research.

Fresh leaves of *D. chinensis* were collected from Haiyuan (Zhongwei, Ningxia, China; coordinates: 105°37′E, 36°12′N) and dried with silica gel. The voucher specimen was stored in Sichuan University Herbarium with the number QTPLJQ14383118. Total genomic DNA was extracted with a modified CTAB method (Doyle and Doyle 1987) and a 350-bp library was constructed. This library was sequenced on the Illumina NovaSeq 6000 system with 150 bp paired-end reads. We obtained 10 million high-quality pair-end reads for *D. chinensis*, and after removing the adapters, the remaining reads were used to assemble the complete chloroplast genome by NOVOPlasty (Dierckxsens et al. 2017). The complete chloroplasts genome sequence of *D. longicalyx* was used as a reference. Plann v1.1 (Huang and CronK 2015) and Geneious v11.0.3 (Kearse et al. 2012) were used to annotate the chloroplasts genome and correct the annotation.

The total plastome length of *D. chinensis* (MT712072) is 149,570 bp, exhibits a typical quadripartite structural organization, consisting of a large single-copy (LSC) region of 82,981 bp, two inverted repeat (IR) regions of 24,748 bp and a small single-copy (SSC) region of 17,093 bp. The cp genome contains 126 complete genes, including 84 protein-coding genes (84 PCGs), 8 ribosomal RNA genes (8 rRNAs), and 34 tRNA genes (34 tRNAs). Most genes occur in a single copy, while 15 genes occur in double, including all rRNAs (4.5S, 5S, 16S, and 23S rRNA), 7 tRNAs (*trnA-UGC*, *trnI-CAU*, *trnI-GAU*, *trnL-CAA*, *trnN-GUU*, *trnR-ACG*, and *trnV-GAC*), and 4 PCGs (*rps*7, *ndh*B, *ycf*2, *rpl*2). The overall GC content of cp DNA is 36.3%, the corresponding values of the LSC, SSC, and IR regions are 34.0%, 29.8%, and 42.5%, respectively.

In order to further clarify the phylogenetic position of *D. chinensis*, plastome of nine representative *Dianthus* species were obtained from NCBI to reconstruct the plastome phylogeny, with *Psammosilene tunicoides* as an outgroup. All the sequences were aligned using MAFFT v.7.313 (Katoh and Standley 2013) and maximum likelihood phylogenetic analyses were conducted using RAxML v.8.2.11 (Stamatakis 2014) under GTRCAT model with 500 bootstrap replicates. The phylogenetic tree shows that *Dianthus* were divided into two subclades. *Dianthus caryophyllus*, *D. moravicus,* and *D. gratianopolitanus* cluster together. Other species cluster in another clade, while *D. chinensis* is a sister to *D. longicalyx* (Figure 1).

**Figure 1.**
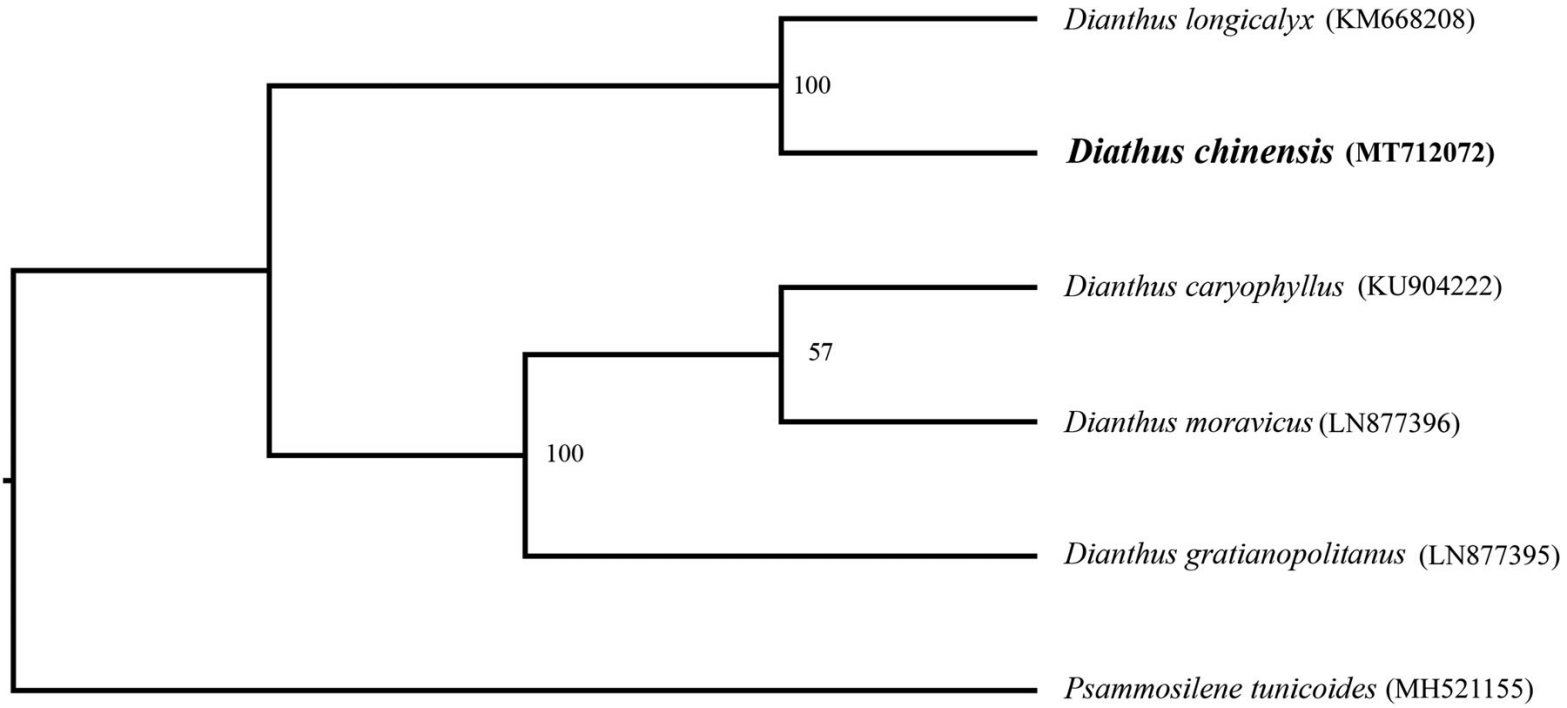
Phylogenetic relationships of *Dianthus* species using whole chloroplast genome. GenBank accession numbers: *Dianthus caryophyllus* (KU904222), *Diathus chinensis* (MT712072), *Dianthus gratianopolitanus* (LN877395), *Dianthus longicalyx* (KM668208), *Dianthus moravicus* (LN877396), *Psammosilene tunicoides* (MH521155).

## Data Availability

The data that support the findings of this study are openly available in GenBank of NCBI at https://www.ncbi.nlm.nih.gov, reference number MT712072.

## References

[CIT0001] Czerepanov SK. 1973. Floram URSS (tomia I-XXX). Addita-menla et Corrigenda ad. Leningrad: Science Press; p. 153–179.

[CIT0002] Dierckxsens N, Mardulyn P, Smits G. 2017. NOVOPlasty: *de novo* assembly of organelle genomes from whole genome data. Nucleic Acids Res. 45(4):e18. 28204566 10.1093/nar/gkw955 PMC5389512

[CIT0003] Doyle JJ, Doyle JL. 1987. A rapid DNA isolation procedure for small quantities of fresh leaf tissue. Phytochem Bull. 19:11–15.

[CIT0004] Huang DI, Cronk QCB. 2015. Plann: a command-line application for anno-tating plastome sequences. Appl Plant Sci. 3(8):1500026. 10.3732/apps.1500026 PMC4542940 26312193

[CIT0005] Katoh K, Standley DM. 2013. MAFFT multiple sequence alignment software version 7: improvements in performance and usability. Mol Biol Evol. 30(4):772–780. 23329690 10.1093/molbev/mst010 PMC3603318

[CIT0006] Kearse M, Moir R, Wilson A, Stones-Havas S, Cheung M, Sturrock S, Buxton S, Cooper A, Markowitz S, Duran C, et al. 2012. Geneious basic: an integrated and extendable desktop software platform for the organization and analysis of sequence data. Bioinformatics. 28(12):1647–1649. 22543367 10.1093/bioinformatics/bts199 PMC3371832

[CIT0007] Shin IS, Lee MY, Ha H, Jeon WY, Seo CS, Shin HK. 2012. *Dianthus superbus* fructus suppresses airway inflammation by downregulating of inducible nitric oxide synthase in an ovalbumin-induced murine model of asthma. J Inflamm. 9(1):41. 10.1186/1476-9255-9-41 PMC3551699 23110404

[CIT0008] Stamatakis A. 2014. RAxML version 8: a tool for phylogenetic analysis and post-analysis of large phylogenies. Bioinformatics. 30(9):1312–1313. 24451623 10.1093/bioinformatics/btu033 PMC3998144

[CIT0009] Tang CL, Lu DQ. 1996. Flora of China. Beijing: Science Press; Vol. 26, p. 47–448.

[CIT0010] Wang YC, Tan NH, Zhou J, Wu HM. 1998. Cyclopeptides from *Dianthus superbus*. Phytochemistry. 49(5):1453–1456.

